# Striking at Survivin: YM-155 Inhibits High-Risk Neuroblastoma Growth and Enhances Chemosensitivity

**DOI:** 10.3390/cancers17193221

**Published:** 2025-10-02

**Authors:** Danielle C. Rouse, Rameswari Chilamakuri, Saurabh Agarwal

**Affiliations:** Department of Pharmaceutical Sciences, College of Pharmacy and Health Sciences, St. John’s University, New York, NY 11439, USA

**Keywords:** neuroblastoma, YM-155, survivin, pediatric cancer, MYCN

## Abstract

Background/Objectives: Neuroblastoma (NB) is an aggressive pediatric malignancy that accounts for nearly 15% of all childhood cancer-related deaths, with high-risk cases showing a poor 20% prognosis and limited response to current therapies. Survivin, encoded by the *BIRC5* gene, is an anti-apoptotic protein frequently overexpressed in NB and linked to treatment resistance and unfavorable clinical outcomes. Methods and Results: An analysis of 1235 NB patient datasets revealed a significant association between elevated *BIRC5* expression and reduced overall and event-free survival, highlighting survivin as an important therapeutic target in NB. To explore this strategy, we evaluated the efficacy of YM-155, a small-molecule survivin inhibitor, across multiple NB cell lines. YM-155 displayed potent cytotoxic activity in six NB cell lines with IC_50_ values ranging from 8 to 212 nM and significantly inhibited colony formation and 3D spheroid growth in a dose-dependent manner. Mechanistic analyses revealed that YM-155 downregulated survivin at both mRNA and protein levels, induced apoptosis by about 2–7-fold, and caused G0/G1 phase cell cycle arrest. Moreover, YM-155 treatment enhanced p53 expression, suggesting reactivation of tumor suppressor pathways. Notably, combining YM-155 and the chemotherapeutic agent etoposide resulted in synergistic inhibition of NB growth with ED75 values ranging from 0.17 to 1, compared to either agent alone. In the xenograft mouse model, YM-155 inhibited tumor burden in contrast to controls by about 3-fold, and without any notable toxic effects in vivo. Conclusion: Overall, our findings identify YM-155 as a promising therapeutic agent for high-risk NB by directly targeting survivin and enhancing chemosensitivity. These results support continued preclinical development of survivin inhibitors as part of rational combination strategies in pediatric cancer treatment.

## Simple Summary

Neuroblastoma (NB) remains one of the most aggressive pediatric cancers, with high-risk disease showing poor prognosis and limited therapeutic options. Survivin (BIRC5), an anti-apoptotic protein frequently overexpressed in NB, is associated with resistance and adverse clinical outcomes. Analysis of NB patient datasets confirmed that high *BIRC5* expression correlates with reduced survival. To investigate survivin targeting, we evaluated YM-155, a small-molecule inhibitor, in NB models. YM-155 demonstrated potent cytotoxicity, suppressed colony formation and 3D spheroid growth, downregulated survivin, induced apoptosis, and caused G0/G1 cell cycle arrest. Combination of YM-155 with etoposide produced synergistic activity, and in vivo YM-155 significantly reduced tumor burden without observed toxicity. These findings establish YM-155 as a promising therapeutic candidate and support survivin inhibition as a rational strategy in NB.

## 1. Introduction

Neuroblastoma (NB) is the most common malignancy diagnosed during the first year of life, accounting for approximately 15% of all childhood cancer-related deaths [1,2]. This embryonal tumor arises from the extracranial sympathetic nervous system and is characterized by high clinical and biological heterogeneity [3,4]. Despite advances in multimodal therapy, high-risk NB remains largely incurable, with survivors often experiencing long-term adverse effects due to the toxicity of conventional treatments. Thus, there is a critical need to identify molecular targets and signaling pathways that drive NB pathogenesis in order to develop more specific, effective, and less toxic therapies [5,6].

The tumor suppressor p53 is a nuclear phosphoprotein that plays a central role in maintaining genomic integrity. While p53 is typically expressed at low levels under normal conditions, it accumulates in response to various cellular stresses, including DNA damage and oncogenic signaling [7]. These stress signals are transmitted through distinct pathways, which reflect the dual role of p53 as both a “guardian of the genome” and a “policeman of the oncogenes” [8]. Interestingly, replication stress can lead to a reversal of the typical p53-dependent inhibition of survivin expression, whereby p53 becomes essential for survivin induction. Survivin, in turn, facilitates mitotic regulation and cellular repair. However, under conditions of persistent or severe damage, p53 can reassert its tumor-suppressive function by triggering apoptosis and preventing malignant transformation [9].

Survivin, also known as baculoviral inhibitor of apoptosis repeat-containing 5 (BIRC5), is a 142-amino acid protein composed of an N-terminal Zn^2+^-binding BIR domain and a C-terminal α-helix motif [10]. These domains mediate the role of survivin in inhibiting apoptosis and regulating mitosis. The C-terminal region also enables interactions with the cytoskeleton via a microtubule-binding site. As the smallest member of the inhibitor of apoptosis (IAP) family, survivin (16.5 kDa) is a multifunctional protein that promotes cell survival, prevents both apoptotic and autophagic death, and can participate in extracellular signaling via exosomes secreted by cancer cells [11]. Survivin is largely absent from most normal adult tissues but is highly expressed in many malignancies. Therefore, it has emerged as an attractive target for anticancer therapy [9]. Survivin overexpression is strongly correlated with poor prognosis and reduced survival in various cancers, including oral, breast, and colorectal carcinomas [12,13].

YM-155 (Sepantronium Bromide) is a small-molecule survivin inhibitor originally designed to inhibit *BIRC5* transcription [9]. This imidazolium-based molecule was organically synthesized by Astellas Pharma, Inc. (Tokyo, Japan) , with a chemical formula of C_20_H_19_BrN_4_O_3_ [14]. In NB models, YM-155 has been shown to reduce cell viability at clinically relevant concentrations by depleting survivin [15]. YM-155 exhibits multiple mechanisms of action, including suppression of survivin at both mRNA and protein levels, synergizes with microtubule-targeting agents [16], enhances radiosensitivity by converting radiation-induced senescence into apoptosis [17], inhibits topoisomerase (Topo) activity leading to DNA damage [18], and induces both apoptosis and mitotic arrest [19]. YM-155 has advanced to Phase I/II clinical trials for several malignancies, including melanoma (NCT00281541), leukemia (NCT01023386), lymphoma (NCT01007292), breast cancer (NCT01038804), and non-small cell lung cancer (NSCLC) (NCT01100931) [20,21]. As a monotherapy, it has demonstrated modest clinical activity with a manageable safety profile across various solid tumors [21,22].

In the present study, we investigated the therapeutic potential of YM-155 in high-risk NB. We demonstrate that YM-155 significantly inhibits NB cell proliferation, colony formation, and 3D spheroid growth in vitro, and significantly reduces tumor burden in vivo. Mechanistically, YM-155 downregulates survivin, enhances apoptosis, and induces cell cycle arrest. Notably, YM-155 also increases p53 expression, suggesting reactivation of tumor-suppressive signaling. In addition, YM-155 sensitizes NB cells to etoposide (VP-16), a chemotherapeutic agent commonly used in combination regimens to treat pediatric solid tumors, including NB [23,24]. Etoposide is a topoisomerase II (Topo2) inhibitor that induces DNA strand breaks and exerts maximal cytotoxicity during the G2/M phase of the cell cycle [25].

Taken together, our study establishes survivin as a critical driver of therapeutic resistance in high-risk NB and validates YM-155 as a potent survivin inhibitor with strong translational potential. Beyond suppressing survivin expression, YM-155 reactivates p53 tumor suppressor pathways, induces apoptosis, enforces cell-cycle arrest, and markedly sensitizes tumors to etoposide. Importantly, the combination of YM-155 with conventional chemotherapy offers a novel, less toxic, and more effective dual-modality treatment strategy for NB. Overall, this study defines survivin inhibition as a rational and clinically actionable therapeutic approach for high-risk pediatric NB.

## 2. Materials and Methods

### 2.1. Drugs and Antibodies

YM-155 was purchased from MedChem Express, Monmouth Junction, NJ, USA, and etoposide (VP-16) from Fisher Scientific, Chicago, IL, USA. Primary antibodies against Survivin, p53, β-actin, and HRP-conjugated secondary antibodies were obtained from Cell Signaling Technology, Danvers, MA, USA.

### 2.2. Cell Culture

Eight NB cell lines were used in this study. Six of the established NB cell lines, including three MYCN-non-amplified (SH-SY5Y, SK-N-AS, CHLA-255) and three MYCN-amplified (NGP, LAN-5, IMR-32), were cultured as previously described [26,27]. Additionally, two patient-derived xenograft (PDX)-derived NB cell lines, COG-N-415 and COG-N-269, were obtained from the Children’s Oncology Group (COG) repository. These PDX lines were maintained in Iscove’s Modified Dulbecco’s Medium (IMDM; Thermo Fisher Scientific, Carlsbad, CA, USA) supplemented with 20% fetal bovine serum, 1% penicillin–streptomycin, 1% L-glutamine, and 1% insulin–transferrin–selenium supplement.

### 2.3. Patient Datasets

A total of 1235 primary NB patient samples were analyzed using the Versteeg (N = 88), Kocak (N = 649), and SEQC (N = 498) datasets accessed via the R2 Genomics Analysis and Visualization Platform. These publicly available datasets contain microarray profiles and associated clinical outcomes, enabling multi-parametric analysis of *BIRC5* expression and survival.

### 2.4. Cell Viability, Clonogenic, and 3D Spheroid Assays

Cell viability assays using the MTT dye and clonogenic assays were conducted as previously described, following standard protocols [28]. Briefly, for cell viability assays, NB cells were seeded in 96-well plates and treated with different concentrations of YM-155 for 72 h. MTT dye was added, and absorbance was measured at 560 nm using a microplate reader (SpectraMax iD3, Molecular Devices, San Jose, CA, USA). IC_50_ values were determined using GraphPad Prism 10 software version 10. For clonogenic assays, NB cells (2.5 × 10^3^/well) were seeded in 6-well plates, treated with YM-155 for 48 h, and then cultured in fresh media for 10–12 days. Colonies were stained with 0.2% crystal violet, imaged, and quantified using ChemiDoc XRS+ (Bio-Rad, Hercules, CA, USA).

For 3D spheroid assays, NB cells were seeded into ultra-low attachment 96-well plates (4515; Corning, Somerville, MA, USA) to form spheroids, as per manufacturer’s recommendations. YM-155 was applied for 12 days with replenishment every three days, and spheroid images were captured. Viability of the spheroids was assessed using Live/Dead Cell Staining (3002; Biotium Inc., Fremont, CA, USA) and CellTiter-Glo^®^ 3D assays (G9683; Promega, Fitchburg, WI, USA) as per manufacturers’ instructions and as described previously [28].

### 2.5. Apoptosis and Cell Cycle Analysis

Apoptosis was measured by eBioscience Annexin V-FITC/PI Apoptosis Detection Kit (BMS500FI-300, Thermo Fisher Scientific, CA, USA), and cell cycle was analyzed using the Click-iT™ EdU Flow Cytometry Assay Kit (C10632, Thermo Fisher Scientific, Santa Clara, CA, USA). Both assays were performed as recommended by the manufacturer and described previously [29]. Cells were analyzed using the Attune NxT Acoustic Focusing Cytometer (Life Technologies, Carlsbad, CA, USA).

### 2.6. RNA Isolation and qRT-PCR

Total RNA was extracted using the RNeasy Plus Mini Kit (Qiagen, Hilden, Germany), and cDNA was synthesized using the High-Capacity cDNA Reverse Transcription Kit (Thermo Fisher Scientific, Santa Clara, CA, USA). qRT-PCR was performed for *BIRC5*, *TP53*, *BCL-2*, *NOXA*, and *PUMA* using SYBR Green Master Mix on a QuantStudio 3 system (Thermo Fisher Scientific, Santa Clara, CA, USA) as described previously [28]. *GAPDH* was used as the internal control. Primers used in this study are listed in Table S1.

### 2.7. Immunoblotting

Western blotting was conducted as previously described [28]. Briefly, protein extracts were resolved by SDS-PAGE, transferred to PVDF membranes, and probed with primary antibodies against survivin and p53, followed by HRP-conjugated secondary antibodies. Bands were visualized using the ChemiDoc XRS+ system (Bio-Rad, Hercules, CA, USA).

### 2.8. Drug Synergy Analysis

Drug interactions between YM-155 and etoposide (VP-16) were evaluated using the Chou–Talalay method implemented through CompuSyn software, version 1.0 [30]. Combination Index (CI) values were calculated to quantify drug interaction effects, where CI **< 1** indicates synergism, CI = 1 denotes an additive effect, and CI > 1 reflects antagonism. In addition, Dose Reduction Index (DRI) values were determined to assess the potential for dose minimization in combination regimens. A DRI > 1 indicates a favorable dose reduction for one or both agents, whereas a DRI < 1 suggests an unfavorable reduction.

### 2.9. In Vivo Xenograft Model

Six-week-old Nu/Nu athymic mice were obtained from Taconic Biosciences and acclimatized for one week under standard housing conditions. Subcutaneous NB xenografts were established by injecting 2 × 10^6^ LAN-5 cells suspended in a 1:1 mixture with Cultrex^®^ Basement Membrane Extract (R&D Systems, Minneapolis, MN, USA) into the lower right flank of mice. Tumor growth was monitored using digital vernier calipers, and volume was calculated using the standard formula: tumor volume = 0.5 × (length × width^2^). Once tumors reached approximately 4 mm in diameter, mice were randomized into two treatment groups (n = 6 per group) and administered either YM-155 (5 mg/kg in 6%PEG, 6% Tween-20 in PBS) or vehicle control every other day for 21 days. Tumor volume and body weight were recorded every three days. At the study endpoint, mice were euthanized, and tumors were excised, weighed, photographed, and processed for downstream analysis. All animal procedures were approved by the Institutional Animal Care and Use Committee (IACUC) of St. John’s University.

### 2.10. Statistical Analysis

All experiments were conducted with at least three biological replicates and two technical repeats. Results are expressed as mean ± SD. Statistical significance was determined using two-tailed Student’s *t*-tests (*p* < 0.05). Kaplan–Meier survival curves and log-rank tests were used for survival analyses.

## 3. Results

### 3.1. BIRC5 Expression Is Associated with Poor Prognosis and NB Progression

**Figure 1:**
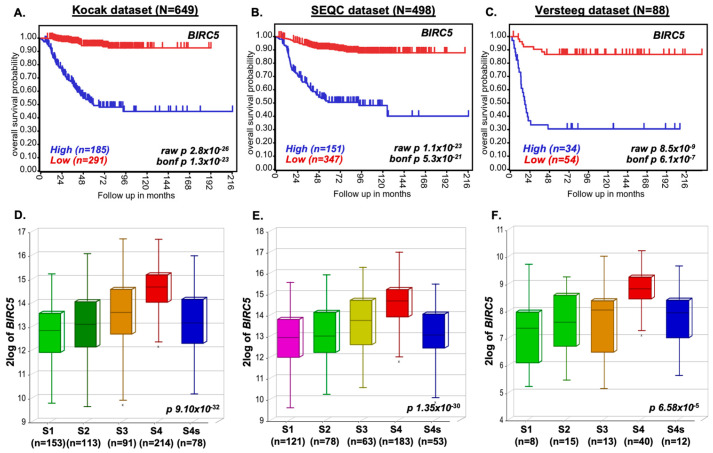
High BIRC5 expression correlates with poor prognosis and advanced disease in NB. (**A**–**C**) Kaplan–Meier survival curves and (**D**–**F**) INSS stage-wise box plots based on *BIRC5* expression in NB tumors. Data derived from three public datasets: (**A**,**D**) Kocak (N = 649), (**B**,**E**) SEQC (N = 498), and (**C**,**F**) Versteeg (N = 88). Elevated *BIRC5* levels are associated with lower survival and higher INSS tumor stage.

To investigate the clinical relevance of *BIRC5* in NB, we analyzed gene expression data from 1235 primary NB patient samples using the R2 Genomics Platform. Kaplan–Meier survival analysis across the Kocak (N = 649), SEQC (N = 498), and Versteeg (N = 88) datasets demonstrated that high *BIRC5* expression was significantly associated with reduced overall survival (Kocak *p* = 1.3 × 10^−23^; SEQC *p* = 5.3 × 10^−21^; Versteeg *p* = 6.1 × 10^−7^) (Figure 1A–C). Further stratification by disease stage showed that advanced-stage NB (INSS stage 4 and 4S) exhibited markedly elevated *BIRC5* expression compared to early-stage tumors (Figure 1D–F). Consistently, *BIRC5* expression was significantly higher in MYCN-amplified tumors across all datasets (Supplementary Figure S1A–C), reinforcing its association with aggressive disease. Collectively, these data highlight *BIRC5* as a potential prognostic biomarker and driver of high-risk NB.

### 3.2. Survivin Inhibition Potently Inhibits NB Proliferation

**Figure 2:**
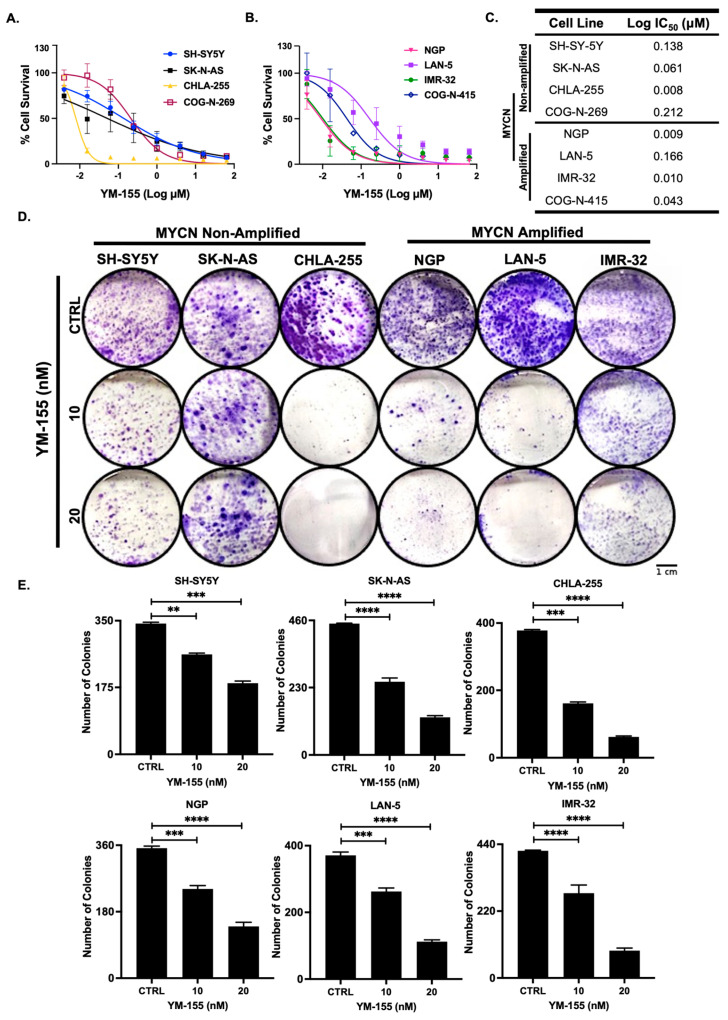
YM-155 suppresses NB cell proliferation and colony formation. (**A**,**B**) Cell viability assessed by MTT assay in MYCN-non-amplified, MYCN-amplified, and patient-derived NB cell lines after 72 h YM-155 treatment. (**C**) Summary table showing IC_50_ values across eight NB lines. (**D**) Representative crystal violet-stained colonies following YM-155 exposure after 48 h. (**E**) Quantification of colony formation. ** *p* < 0.01, *** *p* < 0.001, **** *p* < 0.0001.

To functionally evaluate the therapeutic potential of *BIRC5* inhibition, we treated a panel of NB cell lines, including MYCN-amplified, MYCN-non-amplified, and PDXs, with increasing doses of YM-155. Cytotoxicity assays revealed potent, dose-dependent inhibition of cell viability across all cell lines, with IC_50_ values ranging from 8 to 212 nM (Figure 2A–C). Notably, CHLA-255 and NGP cells exhibited the highest sensitivity with IC_50_ values of 8–9 nM. Further, colony formation assays indicated that YM-155 impaired clonogenic capacity in different NB cell lines in a dose-dependent manner (Figure 2D,E), suggesting a robust anti-proliferative effect through long-term suppression of cell growth.

### 3.3. YM-155 Induces Apoptosis and Arrests Cell Cycle Progression in NB Cells

**Figure 3:**
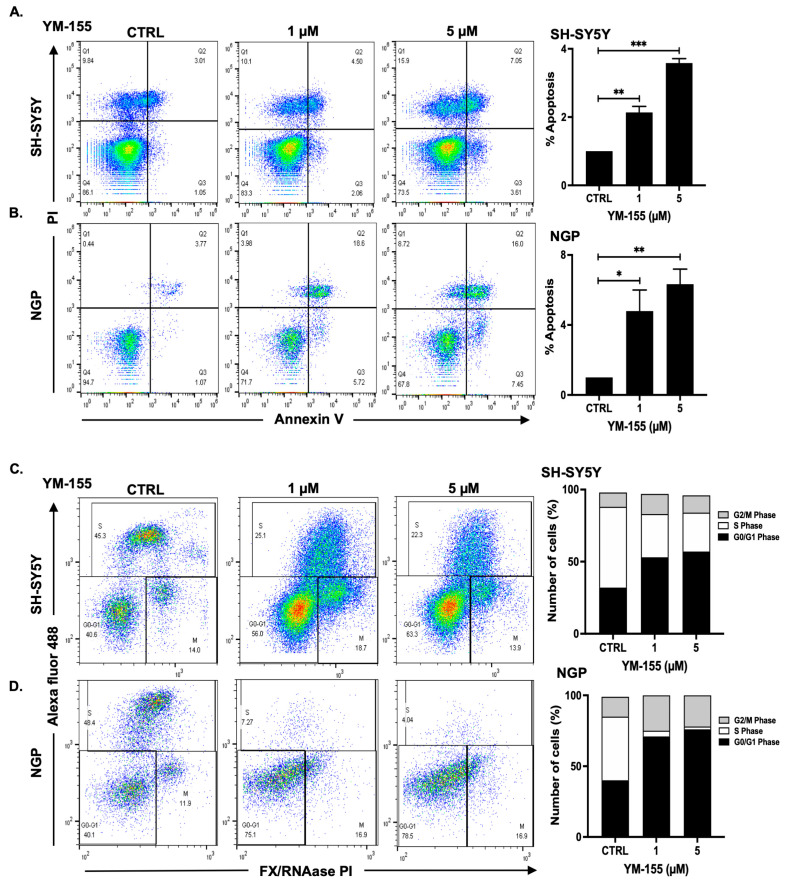
YM-155 induces early apoptosis and cell cycle arrest in NB cells. (**A**,**B**) Flow cytometry analysis of Annexin V/PI-stained SH-SY5Y and NGP cells showing dose-dependent induction of apoptosis in response to YM-155 treatment. (**C**,**D**) Cell cycle distribution after 16 h YM-155 treatment shows accumulation in the G0/G1 phase and a corresponding reduction in the S phase. * *p* < 0.05, ** *p* < 0.01, *** *p* < 0.001.

To determine the mechanism underlying YM-155-mediated NB growth inhibition, we assessed its effects on apoptosis and cell cycle progression. Annexin V/PI staining revealed a dose-dependent increase in early apoptotic cell populations. 5 μM YM-155 treatment resulted in approximately 3.4- and 7.0-fold increases in apoptosis in SH-SY5Y and NGP cell lines, respectively (Figure 3A,B). Flow cytometry-based cell cycle analysis showed that YM-155 treatment led to a significant S phase arrest and corresponding accumulation in the G0/G1. In SH-SY5Y cells, 5 μM YM-155 reduced S-phase cells by 2.0-fold and increased G0/G1-phase cells by 1.6-fold; in NGP cells, a striking 12.0-fold decrease in S-phase and 2.0-fold increase in G0/G1 cells was observed (Figure 3C,D). These findings indicate that YM-155 inhibits NB proliferation by inducing apoptosis and arresting DNA synthesis.

### 3.4. YM-155 Suppresses 3D NB Spheroid Growth and Viability

**Figure 4:**
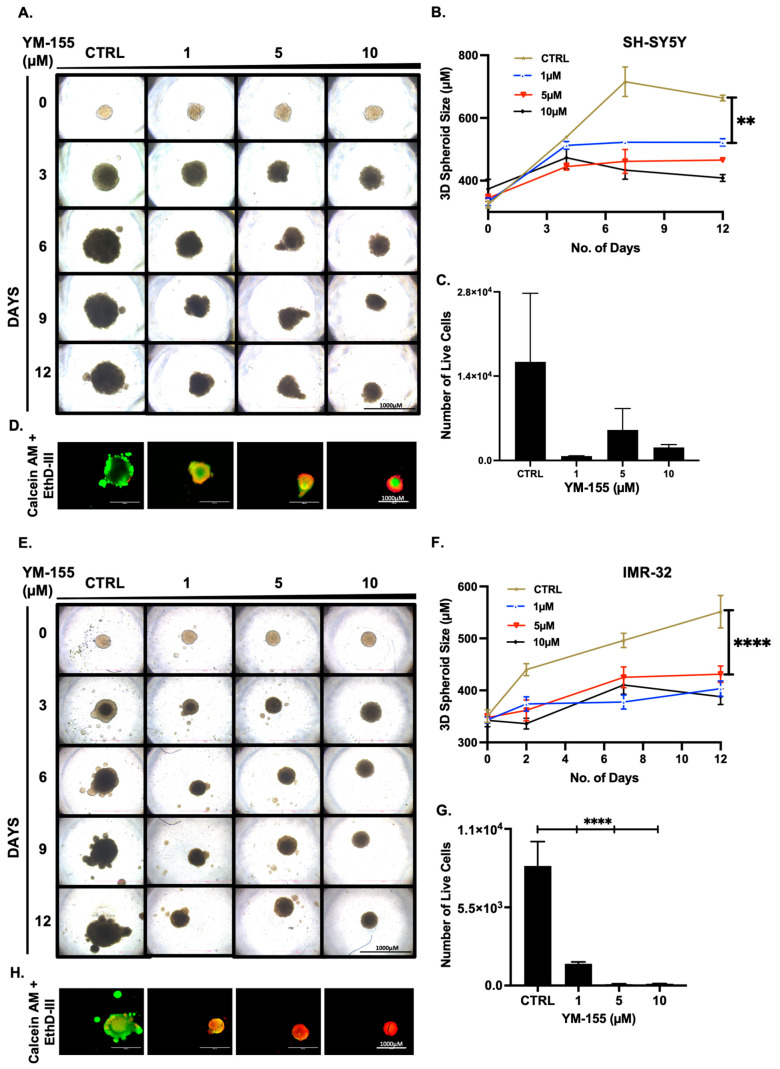
YM-155 inhibits NB 3D spheroid growth. (**A**,**E**) Representative images of SH-SY5Y and IMR-32 spheroids treated with YM-155 over 12 days. (**B**,**F**) Quantification of spheroid growth over time. (**C**,**G**) Live cells quantification on day 12, measuring ATP content using CellTiter-Glo^®^ 3D assay. (**D**,**H**) Representative fluorescence merged images on day 12 using Calcein AM and EthD-III staining. ** *p* < 0.01, **** *p* < 0.0001.

To evaluate YM-155 efficacy under more physiologically relevant conditions, we generated 3D spheroids using SH-SY5Y (MYCN-non-amplified) and IMR-32 (MYCN-amplified) cells. Spheroids were treated with increasing concentrations of YM-155 over 12 days. YM-155 significantly inhibited spheroid size in both models in a dose-dependent manner (Figure 4). Live/dead viability assays revealed that YM-155 induced substantial cell death within spheroids, as evidenced by decreased ATP release (Figure 4C,G) and increased EthD-III fluorescence (Figure 4D,H). These results confirm that YM-155 effectively inhibits the growth of NB tumor-like structures in 3D culture.

### 3.5. YM-155 Inhibits the Survivin Pathway at MRNA and Protein Levels

**Figure 5:**
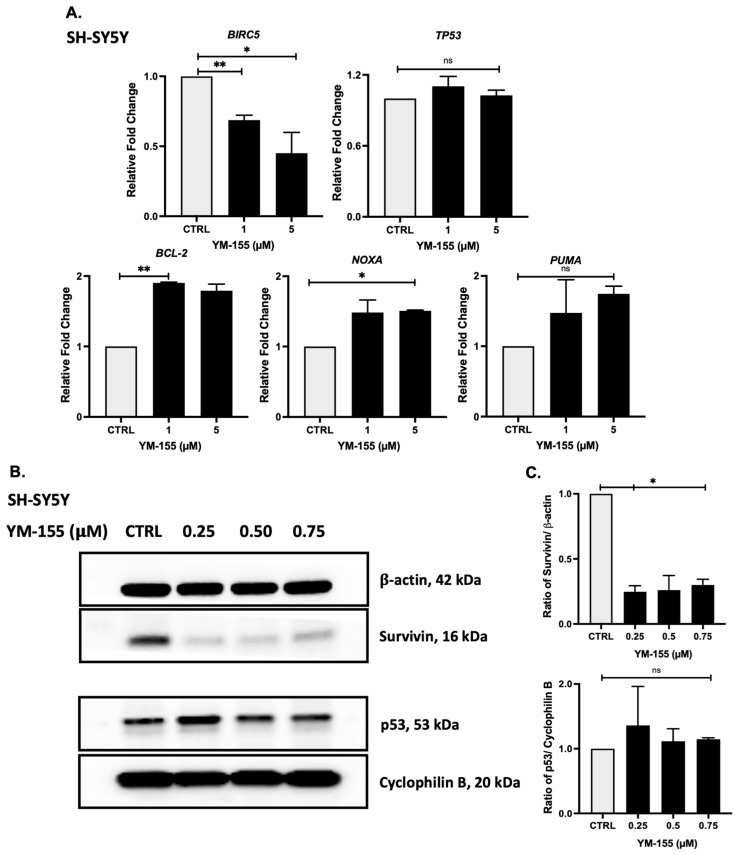
YM-155 inhibits survivin and modulates p53 levels. (**A**) qRT-PCR analysis of *BIRC5*, *TP53*, *BCL2*, *NOXA*, and *PUMA* mRNA levels following YM-155 treatment in SH-SY5Y cells. (**B**) Western blot showing a dose-dependent decrease in survivin protein and increased p53 protein levels. (**C**) Densitometric analysis of survivin expression normalized to β-actin and p53 expression normalized to Cyclophilin B. * *p* < 0.05, ** *p* < 0.01. The original images of the Western Blotting figures can be found in Supplementary Figure S2.

To explore the molecular mechanism of YM-155 action, we assessed expression of apoptosis- and survival-related genes via RT-qPCR in SH-SY5Y cells. YM-155 treatment produced dose-dependent effects on gene expression and significantly downregulated *BIRC5* by more than two-fold at a 5 μM dose, while non-significantly increasing *TP53* expression, and substantially upregulated the anti-apoptotic gene *BCL-2* by two-fold and pro-apoptotic genes *NOXA* and *PUMA* transcripts by 1.5–1.8-fold (Figure 5A).

Western blot analysis demonstrated a dose-dependent reduction in survivin protein levels, showing about a 4-fold reduction with only about 25% of baseline remaining after YM-155 treatment, whereas a modest, non-significant increase in p53 protein levels was also observed. (Figure 5B,C; Supplementary Figure S2). These findings suggest that YM-155 exerts its effects in NB through suppression of survivin and associated apoptotic regulators.

### 3.6. YM-155 Synergizes with Etoposide to Enhance Cytotoxicity in NB Cells

**Figure 6:**
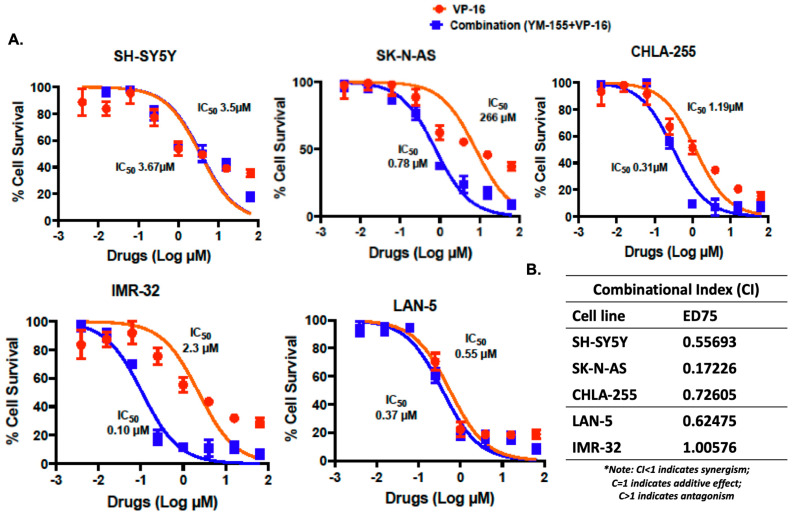
YM-155 sensitizes NB cells to etoposide-induced cytotoxicity. (**A**) Dose–response curves of YM-155 and etoposide in combination across five NB cell lines after 72 h. (**B**) Drug synergy quantified by the Chou–Talalay Combination Index (CI) method using CompuSyn software. CI < 1 indicates synergy.

Given the clinical relevance of etoposide in NB therapy, we tested whether YM-155 enhances etoposide efficacy in treating NB cells. Combination cytotoxicity assays using five NB cell lines revealed that YM-155 potentiated etoposide-induced cell death across both MYCN-amplified and non-amplified lines (Figure 6A). Chou–Talalay analysis confirmed synergism in most lines, with combination index (CI) values at ED75 below 1.0 for four out of five cell lines (Figure 6B; Supplementary Figures S3 and S4). These results indicate that dual targeting of survivin and Topo pathways provides enhanced anti-tumor effects in NB.

### 3.7. Survivin Inhibition Exhibits Potent Anti-Tumor Activity In Vivo

**Figure 7:**
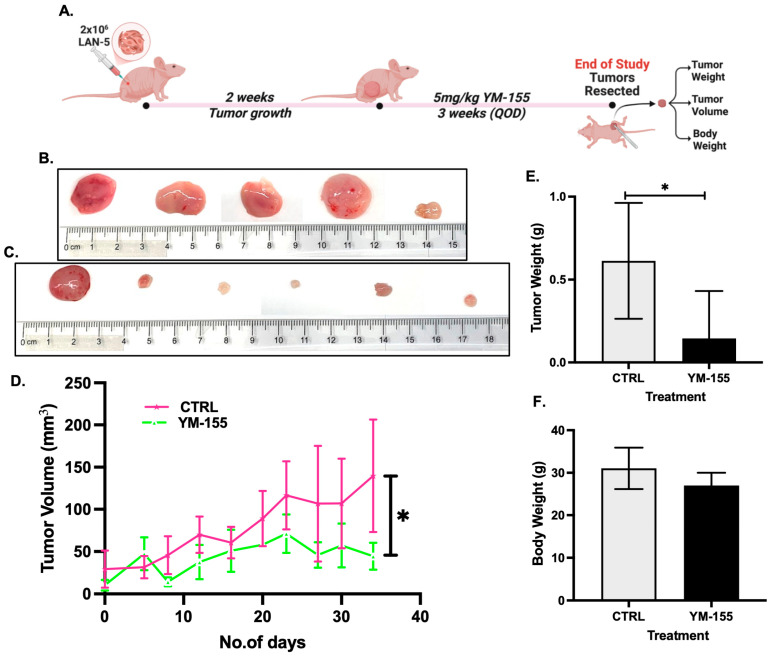
YM-155 inhibits tumor growth in an in vivo NB xenograft model. (**A**) Schematic diagram of the in vivo study. (**B**,**C**) Representative images of LAN-5 tumors in control and YM-155-treated groups. (**D**) Tumor volume quantification in both cohorts. (**E**) Tumor weights at endpoint. (**F**) Body weight monitoring over the treatment period. * *p* < 0.05.

To further validate our findings for YM-155 in an in vivo setting, we developed a subcutaneous xenograft model using LAN-5 NB cells (Figure 7A). Mice treated with YM-155 (5 mg/kg) displayed marked tumor regression compared to controls, with reduced tumor volume and tumor weight at endpoint (Figure 7B–E). Importantly, no significant differences in body weights were observed between groups, indicating minimal systemic toxicity (Figure 7F). These results demonstrate the robust anti-tumor efficacy of YM-155 in vivo, consistent with its survivin-targeted mechanism of action.

## 4. Discussion

High-risk neuroblastoma (NB), often driven by MYCN amplification, remains among the most aggressive pediatric cancers despite intensive multimodal therapy. A critical barrier to improved outcomes is the tumor’s ability to evade apoptosis and resist chemotherapeutics. Survivin (*BIRC5*), a dual-function inhibitor of apoptosis and mitotic regulator, is consistently overexpressed in NB and correlates with disease aggressiveness and poor survival [31,32,33]. By analyzing over 1200 primary NB patient genomic data across three cohorts, we confirmed that elevated *BIRC5* expression stratifies patients into significantly poorer overall and event-free survival groups (Figure 1). These findings mirror recent publications that identify *BIRC5* as a robust prognostic biomarker in pediatric solid tumors [34,35,36].

Our preclinical work demonstrates that YM-155, a small-molecule survivin inhibitor, effectively inhibits NB growth across cellular, spheroid, and xenograft models. This is consistent with several recent reports showing that YM-155 diminishes clonogenic growth and induces apoptosis in both MYCN-amplified and -non-amplified NB lines, and other cancers [37,38,39,40]. Mechanistic dissection revealed that YM-155 induces S-phase cell cycle arrest, reduces clonogenic survival, and triggers apoptosis in a dose-dependent fashion (Figure 2 and Figure 3). These effects were paralleled by downregulation of *BIRC5* and upregulation *of TP53,* anti-apoptotic and pro-apoptotic genes (Figure 5). These molecular responses are consistent with recent mechanistic studies linking YM-155 to disruptions in ubiquitin-specific protease 7 (USP7)-mediated MYCN stabilization as well as induction of replication stress and repressing DNA repair pathways [20,39,40,41,42]. Notably, the upregulation of anti-apoptotic gene *BCL-2* did not affect the overall reduction of tumor regression. The specific mechanism that governs this result is unclear, but previous reports have indicated various pathways, including the combined upregulation of pro-apoptotic *PUMA* and *NOXA,* overcoming the pro-survival of *BCL-2* [43,44,45,46,47].

In 3D spheroid and in vivo LAN5 xenograft models (Figure 4 and Figure 7), YM-155 produced substantial tumor shrinkage without noticeable systemic toxicity, reinforcing its potential clinical translatability. These in vivo effects align with findings in other malignancies where YM-155 reliably reduced tumor burden with minimal adverse effects [38,48,49].

A major novel contribution of this study is the demonstration of synergy between YM-155 and etoposide (VP16). VP-16 is a topoisomerase II inhibitor that elicits DNA double-strand breaks and apoptosis in proliferating tumor cells [50]. In high-risk NB, it is routinely incorporated into induction, consolidation, and salvage regimens [51,52], often in combination with agents such as cisplatin, cyclophosphamide, or ifosfamide [51,53,54], to deepen response. Currently, several clinical trials are exploring novel combinations: for example, NCT04301843 is a Phase II trial evaluating difluoromethylornithine (DFMO) plus etoposide for relapsed or refractory NB; NCT00004110 investigates monoclonal antibody therapy plus etoposide in recurrent NB; and NCT00600132 tests protracted etoposide dosing during induction in high-risk NB. These trials reflect ongoing efforts to enhance etoposide’s therapeutic index and delay or overcome resistance [55]. Our combination index modeling confirmed a synergistic interaction across multiple NB cell lines, suggesting that dual targeting of survivin-mediated survival and Topo2–induced DNA damage potentiates cytotoxicity (Figure 6). Prior studies in adult cancers similarly observed enhanced chemosensitivity when YM-155 was paired with Topo inhibitors [42,56]. Recent investigations have also identified additional off-target mechanisms of YM-155 in other cancers, including mitochondrial damage, AMPK activation, and inhibition of USP-mediated protein stabilization [56,57,58,59]. Additionally, YM-155 directly impairs Topoisomerase II activity [18], provokes DNA damage with phosphorylation of histone H2AX accumulation and PARP hyper-activation [60,61], and downregulates homologous recombination factors including Rad51 [20]. These combined effects provide a mechanistic rationale for the observed synergy between YM-155 and etoposide [56,62,63]. Our findings suggest that these broader impacts may contribute to the cytotoxic efficacy of YM-155 in NB and, consequently, amplify the synergistic effects of the YM-155 and etoposide combinations. These data support further pre-clinical testing of this combination in NB models.

Although our results are compelling, future work should evaluate survivin-directed therapies in orthotopic and PDX models to replicate clinical tumor microenvironments more accurately. Investigating resistance mechanisms, such as altered ABC transporter expression or TP53 status, will inform patient stratification [64]. Furthermore, exploring novel survivin inhibitors may enhance specificity and efficacy [65].

Collectively, our study positions survivin inhibition, via YM-155, as both a molecularly rational and therapeutically actionable strategy for treating high-risk NB. The potent synergy observed with etoposide offers a credible approach for combination therapy, potentially allowing dose reduction and diminishing long-term toxicity. These findings support the need for clinical trials of survivin-targeting regimens, particularly in NB patients with elevated *BIRC5* expression and chemotherapy resistance.

## 5. Conclusions

This study establishes survivin (*BIRC5*) as a critical driver of NB progression and treatment resistance, particularly in high-risk, MYCN-amplified tumors. Using a comprehensive platform of in vitro, 3D spheroid, and in vivo xenograft models, we demonstrate that YM-155, a selective survivin inhibitor, effectively inhibits NB cell proliferation, induces apoptosis, and reduces tumor burden. Notably, YM-155 synergizes etoposide, thereby enhancing chemosensitivity and offering a dual-modality strategy to overcome therapeutic resistance. These findings highlight the translational potential of survivin-targeted therapy and support further preclinical and clinical evaluation of YM-155, alone or in combination with standard chemotherapeutics, as a rational, less toxic approach for improving outcomes in children with high-risk or relapsed NB.

## Data Availability

The raw data supporting the conclusions of this article will be made available by the authors on request.

## Supplementary Materials

The following supporting information can be downloaded at: https://www.mdpi.com/article/10.3390/cancers17193221/s1, Table S1: RT-qPCR primer sequences used for gene expression analysis; Figure S1: Correlation between BIRC5 and MYCN expression in NB datasets; Figure S2: Full western blot images for survivin and p53 expression analysis (corresponding to Figure 5B); Figure S3: YM-155 enhances etoposide efficacy across NB cell lines; Figure S4: YM-155 enables dose reduction of etoposide in NB models.
