# Supplementary material for: Striking at Survivin: YM-155 Inhibits High-Risk Neuroblastoma Growth and Enhances Chemosensitivity
Source: Cancers (Basel). 2025 Oct 2;17(19):3221. doi: 10.3390/cancers17193221 (PMC12524235; doi:10.3390/cancers17193221)
Supplement: Supplementary file 1 [file cancers-17-03221-s001.zip › cancers-3891880-supplementary.pdf]

# Supplementary Materials

## Supplementary Table S1: RT-qPCR Primers used in the study

| Gene | Primer | Sequence |
|---|---|---|
| BIRC5 | survivinB-F<br>survivinB-R | GATGACGACCCCATAGAGGAAC<br>CGCACTTTCTCCGCAGTTT |
| TP53 | TP53-F<br>TP53-R | CAGTTGGGCAGCTGGTTAGG<br>ATCCTCCAGGGTGTGGGATG |
| BCL-2 | BCL-2-F<br>BCL-2-R | GTGGATGACTGAGTACCTGAAC<br>GAGACAGCCAGGAGAAATCAA |
| NOXA | Noxa-F<br>Noxa-R | TACCGCTGGCCTACTGTGAA<br>ATGTGCTGAGTTGGCACTGA |
| PUMA | Puma-F<br>Puma-R | GCGATTGCGATTGGGTGAGA<br>TACTTCCTGCCCTGCTCTGG |
| GAPDH | GAPDH-F<br>GAPDH-R | CACCATCTTCCAGGAGCGAG<br>TGATGACCCTTTTGGCTCCC |

**Supplementary Materials**

**Figure S1**

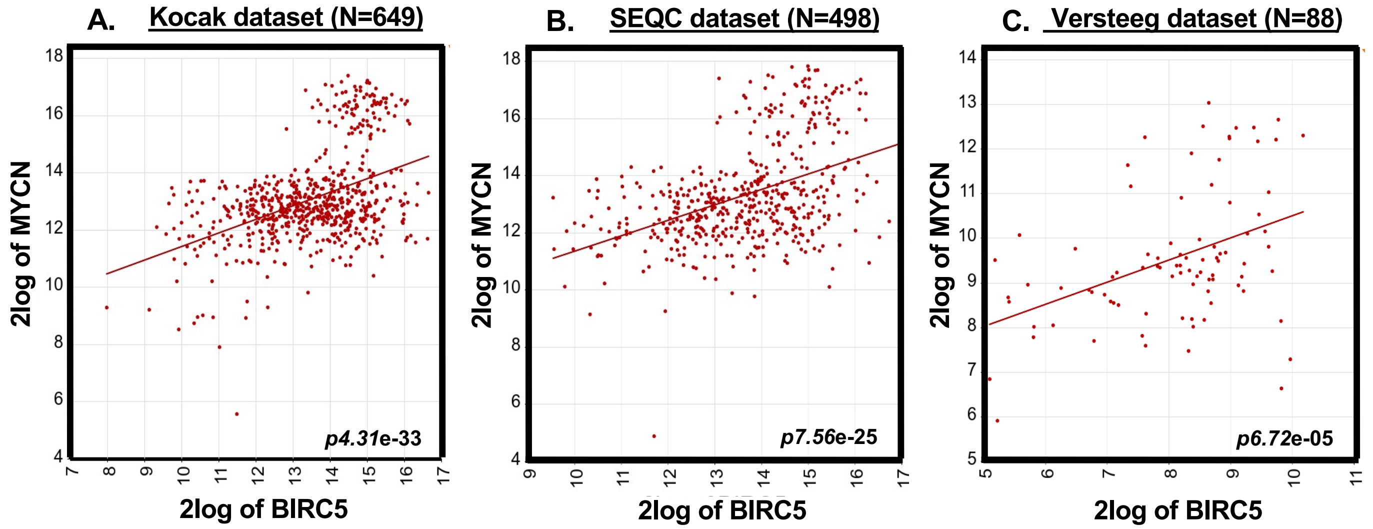


**Figure S1.** *Correlation between BIRC5 and MYCN expression in NB datasets.* XY scatter plots from **(A)** Kocak, **(B)** SEQC, and **(C)** Versteeg datasets show positive correlation between *BIRC5* and *MYCN* expression.

# Figure S2

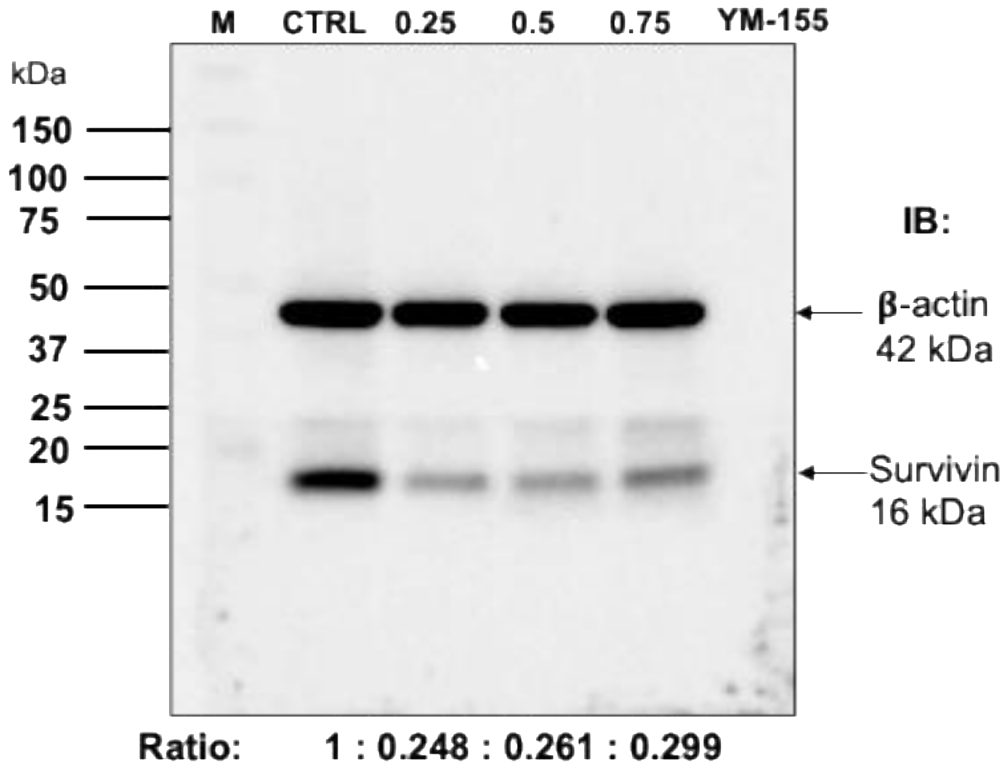


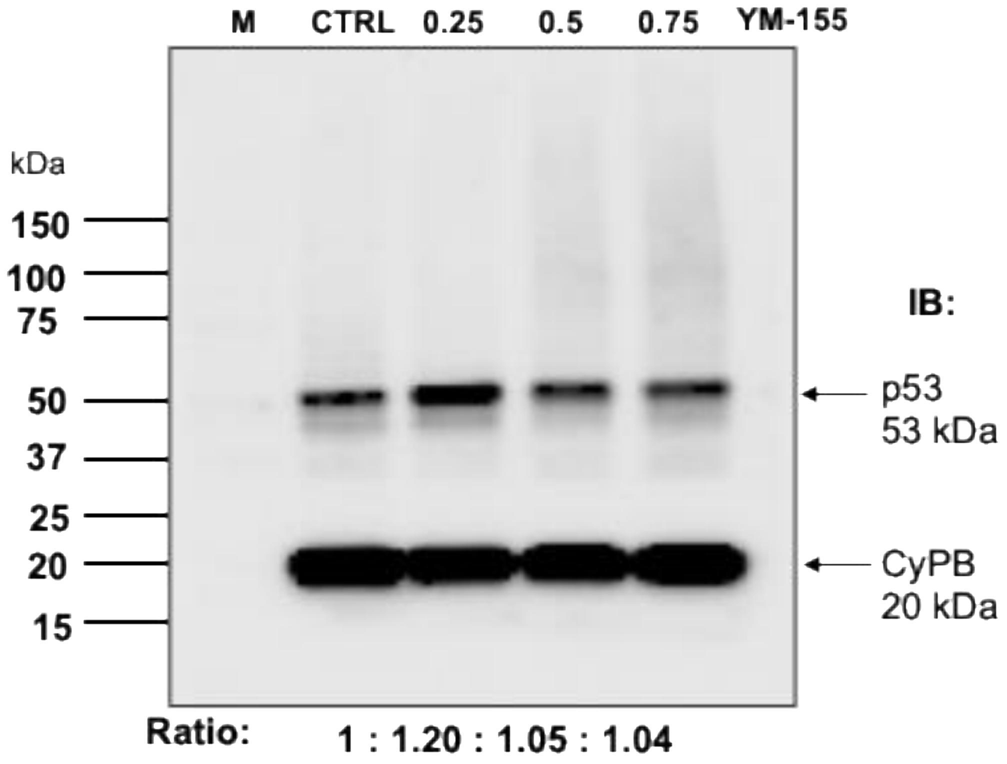


**Figure S2.** Full western blot images and densitometry analysis (corresponding to Figure 5B). M corresponds to the marker lane.

## Supplementary Materials

### Figure S3

**Combination Index Plot**

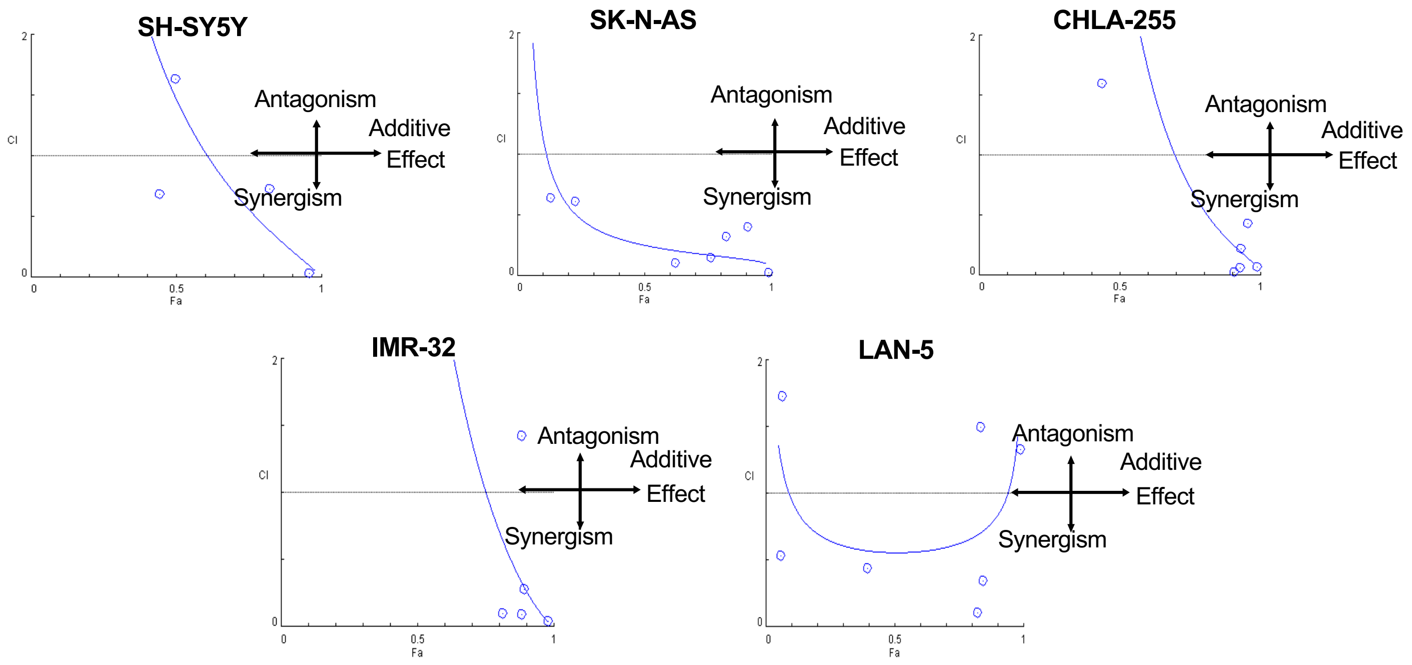


**Figure S3.** *YM-155 enhances etoposide efficacy across NB cell lines.* Combination index plots generated using CompuSyn software for five NB cell lines treated with YM-155 + etoposide for 72 h. CI<1 confirms synergy.

## Supplementary Materials

### Figure S4

### Dose Reduction Index Plot

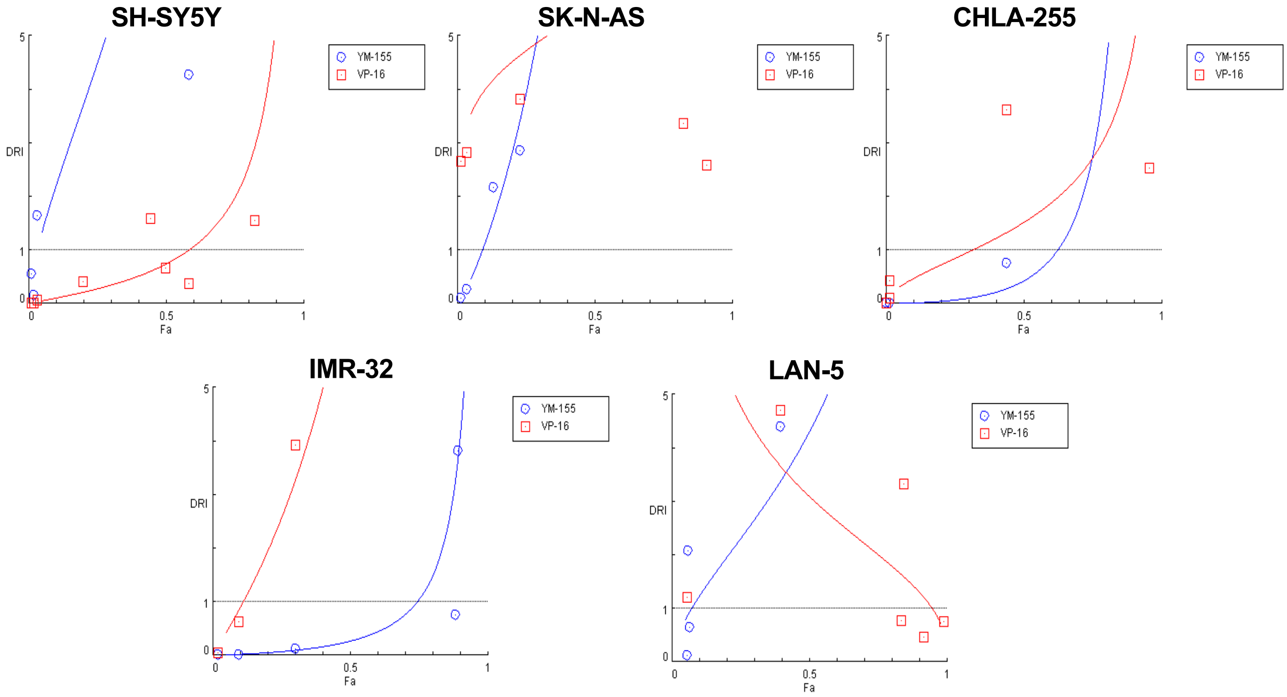


**Figure S4.** *YM-155 enables dose reduction of etoposide in NB models.* Dose reduction index (DRI) plots calculated via the Chou–Talalay method show a favorable DRI (>1) for YM-155 + etoposide, indicating reduced drug burden while maintaining efficacy.
